# Shelf-Life Evaluation of Bilayered Human Skin Equivalent, MyDerm™

**DOI:** 10.1371/journal.pone.0040978

**Published:** 2012-08-23

**Authors:** Wan Tai Seet, Manira Maarof, Khairoji Khairul Anuar, Kien-Hui Chua, Abdul Wahab Ahmad Irfan, Min Hwei Ng, Bin Saim Aminuddin, Bt Hj Idrus Ruszymah

**Affiliations:** 1 Tissue Engineering Centre, Universiti Kebangsaan Malaysia Medical Centre, Kuala Lumpur, Malaysia; 2 Department of Physiology, Faculty of Medicine, Universiti Kebangsaan Malaysia, Kuala Lumpur, Malaysia; 3 Ear, Nose and Throat Consultant Clinic, Ampang Puteri Specialist Hospital, Selangor, Malaysia; University Hospital Hamburg-Eppendorf, Germany

## Abstract

Skin plays an important role in defense against infection and other harmful biological agents. Due to its fragile structure, skin can be easily damaged by heat, chemicals, traumatic injuries and diseases. An autologous bilayered human skin equivalent, MyDerm™, was engineered to provide a living skin substitute to treat critical skin loss. However, one of the disadvantages of living skin substitute is its short shelf-life, hence limiting its distribution worldwide. The aim of this study was to evaluate the shelf-life of MyDerm™ through assessment of cell morphology, cell viability, population doubling time and functional gene expression levels before transplantation. Skin samples were digested with 0.6% Collagenase Type I followed by epithelial cells dissociation with TrypLE Select. Dermal fibroblasts and keratinocytes were culture-expanded to obtain sufficient cells for MyDerm™ construction. MyDerm™ was constructed with plasma-fibrin as temporary biomaterial and evaluated at 0, 24, 48 and 72 hours after storage at 4°C for its shelf-life determination. The morphology of skin cells derived from MyDerm™ remained unchanged across storage times. Cells harvested from MyDerm™ after storage appeared in good viability (90.5%±2.7% to 94.9%±1.6%) and had short population doubling time (58.4±8.7 to 76.9±19 hours). The modest drop in cell viability and increased in population doubling time at longer storage duration did not demonstrate a significant difference. Gene expression for CK10, CK14 and COL III were also comparable between different storage times. In conclusion, MyDerm™ can be stored in basal medium at 4°C for at least 72 hours before transplantation without compromising its functionality.

## Introduction

Skin is the largest and highly complex organ that covers the outer part of human body [1]. Anatomically and functionally, it consists of two layers: the superficial epidermal layer which is mainly made up of keratinocyte and the deeper dermal layer which is made up of fibroblast [2], [3], [4]. The epidermal layer acts as the front line defense against infection and other harmful biological agents [1], [2]. It also plays important roles in regulating body heat, preventing the loss of moisture and nutrients from the body. [5], [6]. In contrast, the dermis layer provides the elasticity and mechanical integrity of the skin [4]. However, due to its soft and fragile structure, skin can be easily damaged under certain circumstances such as heat, chemical, traumatic injuries and certain diseases such as diabetes when the healing ability is significantly diminished. [1].

In clinical practice, wound healing could have two implications. The first represents the property of the body which acts to replace and repair the damaged tissue that can only be feasible in case of minor injuries. This healing process involves regeneration of the epidermis and repair of the dermis, sometimes with the formation of scars.

The second is the intervention of a third party who carries out an active process designed to accelerate or modify the natural healing process in case of major injuries such as extensive burns or non-healing ulcers. The third party intervention is commonly known as surgical intervention which will produce the conditions that will lead to spontaneous wound healing. It can also be the application of biological or biocompatible materials which will act to close the wound and reach a stable and acceptable endpoint to the healing process [7]. The common surgical treatment for major skin loss is the split skin graft (SSG) where a layer of skin is removed from a healthy part of the body (donor site) and grafted onto the wound. Although this is an effective treatment for burn and trauma cases, there are certain disadvantages including the limited availability of graftable skin and the increase risk of infection for patients with massive skin loss. Moreover, the process is painful and may result in scarring, and is not suitable for diabetic individuals with compromised healing abilities. These limitations lead to the use of skin substitutes for the treatment of skin loss caused by major injuries or diseases.

There are three categories of skin substitutes: the first type is grafts made from cultured keratinocytes forming the epidermal layer with no dermal components, the second type is grafts made from cultured fibroblasts forming the dermal layer with no epidermal components, and the third is a bilayered skin graft containing both dermal and epidermal layer [4]. Many skin substitutes are derived from a mixture of biological components and non-biological substances [2] and therefore are inappropriate for clinical applications due to their non-autologous nature. Most of these skin substitutes (Biobrane™ [8], Transcyte™ [2], [9], Apligraf™ [2], [9], Dermagraft™ [10], [11]) are for wound coverage and act as a temporary biological dressing that needs to be removed after a certain period of time [12], [13], [14], [15]. Other skin substitutes such as Alloderm [16] and Integra™ [17], [18] are produced using cultured autologous keratinocytes used for wound closure [4] but the presence of allogenic tissues and animal products, as well as unstable epithelium, causes spontaneous blistering [4].

Due to these shortcomings, a fully autologous tissue engineered bilayered skin substitute named MyDerm™ was constructed using cells harvested from a small piece of skin biopsy from the patient, patient's serum as a culture medium supplement and fibrin from patient's plasma as biomaterial [3]. This fully autologous skin substitute can eliminate the risk of immune rejection and cross contamination. This skin substitute is also a full-thickness bilayered skin. It can be completely taken up and integrated into the patient's skin, and is considered to have great potential to treat massive skin loss. However, there are many criteria that need to be achieved before this skin substitute can be released for clinical application. Among the criteria are the shelf life and the quality of the product after storage. In this study, the quality of MyDerm™ was evaluated by means of cells viability, population doubling time, gene expression level as well as the morphological and histological assessment at different storage period of 0, 24, 48 and 72 hours at 4°C before transplantation to determine the shelf life.

## Materials and Methods

### Ethics Statement

Ethics approval for this research was obtained from Universiti Kebangsaan Malaysia Medical Research and Ethics Committee (UKM-MREC) prior to skin harvesting and blood collection (approval code: FF-069-2003). The research project is compliant to the International Conference of Harmonization (ICH) – Good Clinical Practice Guidelines. Written Informed Consent was obtained from all participants involved in this study. The Informed Consent Form has also been approved by the UKM-MREC prior to commencement of the study.

### Sample Harvesting and Processing

Skin samples from six donors undergoing abdominoplasty or face-lift were obtained with informed consent. The skin tissues were processed within 24 hours from harvest. Blood was also collected from the same donors in plain tubes (for serum collection) and sodium citrate tubes (for plasma collection) (Greiner Bio-One, North Carolina, USA). Serum and plasma were extracted separately by centrifugation at 500×g for 5 minutes at room temperature. The resulting serum and plasma were then filtered through a 0.2 µm filter into new sterile 50 mL collection tubes. Plasma was stored at −20°C for later use in the bilayered skin construction and the serum was used as growth supplement in the culture media.

### Isolation of Keratinocytes, Dermal Fibroblasts and Co-culture

Skin samples of a standard size (1 cm×3 cm) were processed. Fat, hair and debris were removed from the skin samples and swabbed with 70% isopropyl alcohol, rinsed with calcium and magnesium free Dulbecco's Phosphate Buffered Saline (DPBS, GIBCO, NY, USA) supplemented with antibiotics (Gentamycin, GIBCO. Antibiotic-Antimycotic, Gentaur, Belgium) and then cut into smaller pieces of approximately 1 mm×2 mm. This was followed by digestion with 0.6% Collagenase Type I (Worthington, NJ, USA) for 5–6 hours in a 37°C incubator shaker followed by cell dissociation with TrypLE Select (GIBCO) for 20 minutes in a 37°C incubator shaker to obtain both keratinocytes and dermal fibroblasts. The cell suspension was centrifuged for 5 minutes at 600×g and the cell pellets were rinsed with DPBS. The cells were then re-suspended in 6 mL of co-culture medium (mixture of Nutrient Mixture F-12∶Dulbecco's Modified Eagle Medium (at 1∶1 ratio, Sigma Aldrich) containing 10% human serum and Defined Keratinocyte Serum Free Medium (DKSFM, GIBCO, NY, USA) at 1∶1 ratio) and seeded into 3 wells of a six-well culture plate (Nunc, USA) and maintained in a 37°C incubator with 5% CO_2_. The co-culture medium was changed every 48 to 72 hours.

### Differential Trypzinization for Fibroblast and Keratinocyte Culture Separation

When the co-culture has reached 70–80% confluency, the culture medium was removed and the cells were rinsed with DPBS. Following this, 2 mL of TrypLE Select was added into each well and incubated for 5 minutes in a 37°C incubator with 5% CO_2_ to detach only the fibroblasts. The detached fibroblasts were collected and cultured in a T75 flask with F12∶DMEM +10% human serum and the medium was changed every 72 hours. The remaining keratinocytes still attached on the plate were rinsed with DPBS and continued to be cultured in DKSFM with medium change every 48 to 72 hours. Subsequently, upon reaching 70–80% confluency, keratinocytes and fibroblasts were trypsinized with TrypLE Select and subcultured with a seeding density of 1.0×10^5^ cells per well (keratinocytes) or flask (fibroblast) in its respective culture medium until the desired amount of cells were obtained for the formation of the bilayered skin construct.

### Bilayered Skin MyDerm™ Construction

A piece of surgical silk (Boston Medical Products, Massachusetts, USA) was cut to the size of 9.6 cm^2^ and placed into one well of a six-well culture plate under sterile condition. The silk was affixed to the well with a few drops of 10% CaCl_2_ (Calcium Chloride Dihydrate, American Regent, NY, USA). Any excess CaCl_2_ was then removed from the well.

#### A) Fibrin - keratinocyte Layer

The keratinocytes were trypsinized and cell count was performed by trypan blue staining to ensure sufficient amount of cells were acquired (1–2×10^6^ cells). The cell suspension was centrifuged at 700×g for 5 minutes and pellet was then suspended with 2 mL of the donor's autologous plasma and 25 ul 10% CaCl_2_ was added to the mixture to initiate the polymerization process. The admixture was then dispensed quickly into the 9.6 cm^2^ well, on top of the silk, for polymerization to take place in order to form the fibrin-keratinocyte layer.

#### B) Fibrin - fibroblast Layer

The fibroblasts were trypsinized and cell count was performed by trypan blue staining to ensure sufficient amount of cells were acquired (1–2×10^6^ cells). The cell suspension was centrifuged at 700×g for 5 minutes and the pellet was then suspended with 1 mL of the donor's autologous plasma and 12.5 ul 10% CaCl_2_ was added to the plasma mixture to initiate the polymerization process. This admixture was quickly laid on top of the fibrin-keratinocyte layer to form the fibrin-fibroblast layer and complete the final bilayered construct, MyDerm™ (fibrin-fibroblast and fibrin-keratinocyte skin equivalent). MyDerm™ was immersed in 2 mL of basal F12∶DMEM medium (1∶1 ratio without human serum) in the culture plate, sealed with parafilm and stored at 4°C. Humidity control is not necessary.

### Shelf-life Evaluation of MyDerm™ at Different Storage Time

MyDerm™ was stored at 4°C for 24, 48 and 72 hours before being evaluated by cell morphologic feature assessment, viability test and gene expression quantification. The construct at 0 hour was processed immediately as the control group.

### Histological Analysis with H&E Staining

Approximately one quarter of MyDerm™ at 0, 24, 48 and 72 hours storage time were fixed in formalin overnight. The tissues were then processed and embedded in paraffin. Thin sections (5 um) were prepared using a microtome, dewaxed with a series of xylene and alcohol and then evaluated histologically using hematoxylin and eosin staining (H&E staining) at 40× magnification [3].

### Immunohistochemistry Staining

The skin construct were fixed in formalin overnight and embedded in paraffin. Thin tissue sections (5 um) were prepared using a microtome, deparaffinized in xylene and rehydrated in ethanol. The tissue sections were heated at 98°C for 30 minutes for antigen retrieval, washed with TBS for 5 minutes and blocked in 10% goat serum (Sigma-Aldrich) for 20–30 minutes at 37°C. The tissue sections were then incubated with Rabbit Anti-Human Polyclonal Collagen III Primary Antibody (Abcam) and Mouse Anti-Human Cytokeratin 14 Monoclonal Antibody (Milipore) overnight at 4°C. On the following day, the tissue sections were incubated with Alexa Fluor® 594 goat anti-mouse IgG (red-fluorescent dye staining on the keratinocytes) (Invitrogen) and Alexa Fluor® 488 goat anti-rabbit (green-fluorescent dye staining on the fibroblasts) (Invitrogen) for 1 hour at 37°C and counterstained with DAPI (Dako) for 15 minutes. Slides were evaluated and documented using a Nikon A1R Confocal microscope (Nikon, Tokyo).

### Immunocytochemistry Staining

Fibroblasts and keratinocytes were liberated from MyDerm™ and co-cultured in a 6-well tissue culture plate for 144 hours (6 days). The cells were then fixed in 4% paraformaldehyde (Sigma- Aldrich) in the tissue culture plate for 1 hour, permeabilized for 5 minutes with 0.1% Triton X-100 solution (Sigma-Aldrich), and blocked with 10% goat serum (Sigma-Aldrich) for 20–30 minutes at 37°C. The cells were incubated with Rabbit Polyclonal Collagen III Primary Antibody (Abcam) and Mouse anti-cytokeratin 14 monoclonal antibody (Milipore) overnight at 4°C. On the following day, the cells were incubated with Alexa Fluor® 594 goat anti-mouse IgG (red-fluorescent dye staining the keratinocytes) (Invitrogen) and Alexa Fluor® 488 goat anti-rabbit (green-fluorescent dye staining the fibroblasts) (Invitrogen) for 1 hour at 37°C and counterstained with DAPI (Dako) for 15 minutes. Cells were then evaluated and documented using a Nikon Eclipse Ti fluorescence microscope (Nikon, Tokyo).

### MyDerm™ Cell Viability and Growth Kinetics after Storage

Half piece of the MyDerm™ was digested with 0.3% Collagenase Type I for 30–40 minutes in a 37°C incubator shaker. The cell suspension was centrifuged for 5 minutes at 600×g and the cell pellet was rinsed with DPBS before being suspended in co-culture medium and seeded into 3 wells of a six-well culture plate. Co-culture medium was replaced after 72 hours and the cells were trypsinized after 144 hours of culture. Cell morphological was observed and recorded under the phase contrast microscope. Cell counts were performed for the analysis of growth kinetics and viability.

#### A) Cell viability determination

Cell Counting was performed using Trypan Blue (0.4%, Sigma-Adrich) exclusion assay with a Neubauer hemocytometer.

The percentage of viable cells was calculated using the formula below:
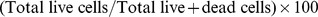



#### B) Growth kinetics determination

The population doubling time (PDT) was used to determine the growth kinetics of fibroblasts and keratinocytes and it was calculated using the formula below:
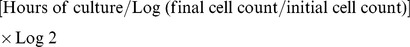


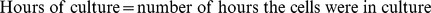


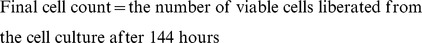


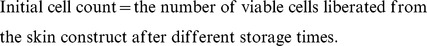



### Quantitative Gene Expression Analysis by Real-Time PCR

#### Total RNA Extraction

Total RNA from approximately one quarter of MyDerm™ was extracted using TRI Reagent (Molecular Research Center, Cincinnati, OH). Polyacryl Carrier (Molecular Research Center) was added in each extraction to precipitate the total RNA. The RNA pellet was then washed with 75% ethanol and dried before being reconstituted in RNAse and DNAse-free distilled water (Invitrogen, Carlsbad, CA). Yield and purity of the isolated RNA was determined by spectrophotometer (Bio-Rad, Hercules, CA). Total RNA was stored at −80°C immediately after extraction.

#### Gene Expression Analysis by RT- PCR

Gene expression level of skin extracellular matrix; type III collagen (COL III), Cytokeratin 10 (CK10) and Cytokeratin 14 (CK14) was quantitatively analyzed with real-time PCR technique. Expression level of each targeted gene was normalized to GAPDH. All primers were designed with Primer3 on-line software (http://frodo.wi.mit.edu) and blasted with GenBank database sequences in order to obtain primers with high specificity. The efficiency and specificity of each primer set was confirmed with standard curve (Ct value versus serial dilution of total RNA) and melting profile evaluation. Primer sequences used in this study are shown in Table 1. Real-time PCR reaction was performed with 100 ng of total RNA, 400 nM of each primer and iScript One-Step RT-PCR kit with SYBR Green (Bio-Rad) according to the manufacturer's instruction. Reactions were run using Bio-Rad iCycler with the following reaction profile: cDNA synthesis for 30 min at 50°C; pre-denaturation for 2 min at 94°C; PCR amplification for 38 cycles with 30 sec at 94°C, 30 sec at 60°C and 30 sec at 72°C. This series of cycles was followed by a melt curve analysis to check the reaction specificity. Expression level of each targeted gene was normalized to GAPDH and was subjected to statistical analysis.

**Table 1:** Primer sequences used in real-time PCR for quantitative gene expression analysis.

Genes	Accession #	Primer 5′ – 3′	PCR Product (bp)
GAPDH	BC 020308	F: 5′-tcc ctg agc tga acg gga ag-3′	217
		R: 5′-gga gga gtg ggt gtc gct gt-3′	
Type III collagen	NM 000090	F: 5′-gtt gac cct aac caa gga tgc a-3′	203
		R: 5′-gga agt tca gga ttg ccg tag-3′	
Keratin 10	NM 000421	F: 5′-gag caa gga act gac tac ag-3′	249
		R: 5′-ctc ggt ttc agc tcg aat ct-3′	
Keratin 14	BC 002690	F: 5′-aga acc gca agg atg ccg ag-3′	150
		R: 5′-act gca gct caa tct cca gg-3′	

Data from Genbank database sequence.

### Statistical Analysis

Results were shown as mean ± SEM. The comparison of mean between groups was accessed with Student's paired t-test and One-Way Analysis of Variance (ANOVA) along with the Bonferroni post hoc multiple comparison test. A P value of less than 0.05 was considered significant.

## Results

### Gross Morphology of Bilayered Skin, MyDerm™

**Figure 1:**
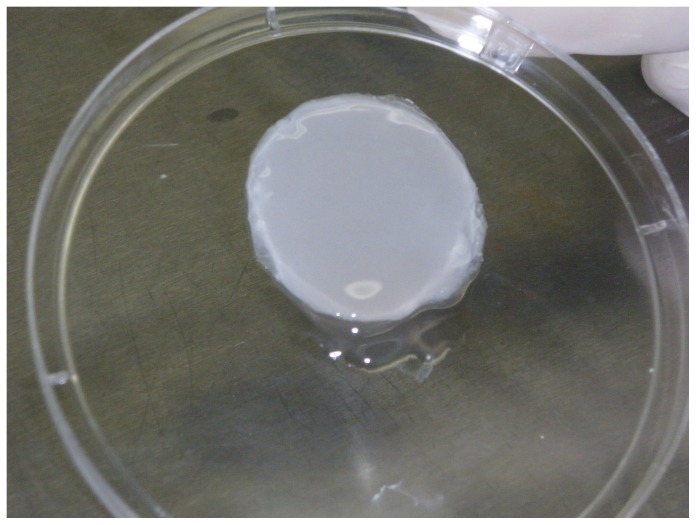
MyDerm™ (9.6 cm^2^ construct) before being processed for analysis.

MyDerm™ appeared opaque and yellowish in color. The construct is soft and fragile and therefore it is important to have the silk layer for physical support. The silk is blue in color to aid in visualization and hence giving a bluish tint to the skin construct as shown in Figure 1. There were no physical changes in MyDerm™ after 72 hours storage at 4°C and the opacity of the construct also remained unchanged overtime.

### Morphology of Fibroblasts and Keratinocytes Liberated From MyDerm™

**Figure 2:**
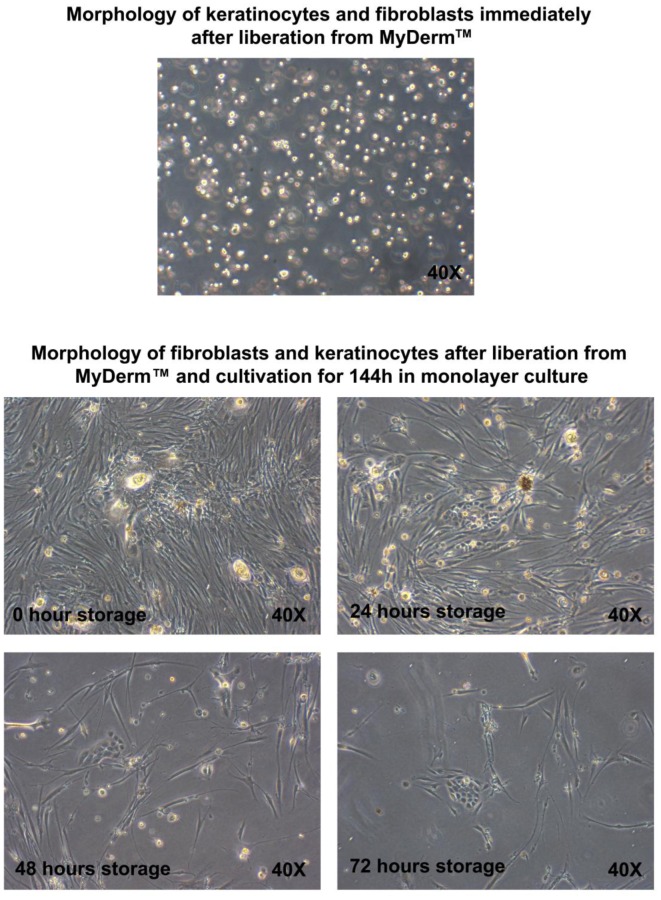
Morphology of fibroblasts and keratinocytes immediately after liberation from MyDerm™ and after cultivation for another 144 h in monolayer culture. Prior to liberation, MyDerm™ was stored for 0, 24, 48, and 72 hours at 4°C. Magnification 40×.

MyDerm™ was incubated for different time storage periods of 0, 24, 48 and 72 hours before liberation of cells and followed by a culture period of 144 hours. In each storage period, both keratinocytes and fibroblasts appeared to be single, round and shining under microscopy observation immediately after liberation from the skin equivalents as shown in Figure 2. It is impossible to differentiate them at this stage. When the cells attached to the culture dish, their morphology changed to polygonal shape for keratinocytes and spindle shape for fibroblasts. After 72 hours, when the culture medium was replaced with fresh medium, those non-attached cells were discarded during media change. After the cells were co-cultured for an additional of 72 hours, the density of the cells increased. The efficiency of cell adherence was judged by comparing the cell density from MyDerm™ of different storage times. Cells became less efficient in adhering to culture plates when construct was stored for longer periods, but the morphology of the cells remained the same compared to the cells from the control group (0 hour storage). The morphology of the fibroblasts remained spindle-shaped and the keratinocyte colonies appeared as groups of polygonal cells. Keratinocytes and fibroblasts were more sparsely distributed in the 72 hours storage group compared to other time storage groups as depicted in Figure 2, an indication of reduced adherence efficiency.

### Histological Analysis of MyDerm™ at Different Storage Time

**Figure 3:**
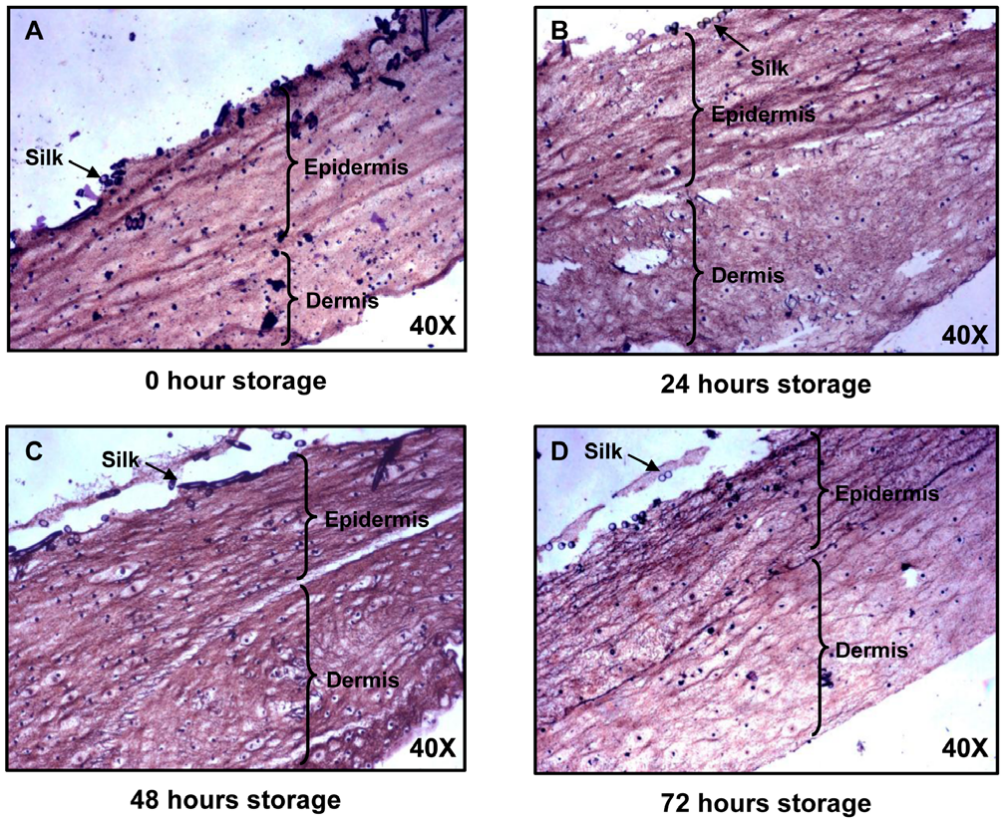
Paraffin section of MyDerm™ at different storage time showing keratinocytes and fibroblasts layers in H&E staining. Magnification 40×.

H&E staining (Figure 3) of MyDerm™ showed homogenous distribution of cells in all the constructs after being stored for different time period. The dark purple/black spots denote cell nucleus. No differences were detected in the histological feature of various time storage groups.

### Immunohistochemistry and Immunocytochemistry Staining

**Figure 4:**
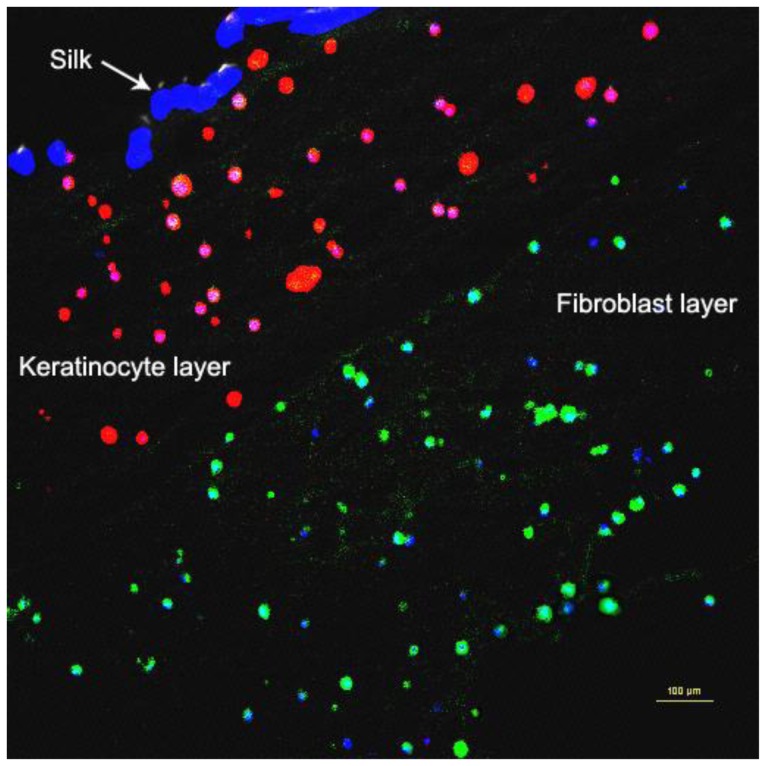
Paraffin section of MyDerm™ at 72 hours storage showing distinct keratinocytes and fibroblasts layers in immunohistochemistry after incubation with Rabbit Anti-Human Polyclonal Collagen III Primary Antibody (green) and Mouse Anti-Human Cytokeratin 14 Monoclonal Antibody (red).

Immunohistochemistry staining (Figure 4) of MyDerm™ with Rabbit Anti-Human Polyclonal Collagen III Primary Antibody (green) and Mouse Anti-Human Cytokeratin 14 Monoclonal Antibody (red) showed that the 3D construct is made up of two distinct layers. The large dark blue patches (silk fibers) at the upper left corner marked the orientation of the skin equivalent with the keratinocytes located immediately beneath the silk layer and fibroblasts located beneath the keratinocytes layer. The smaller blue dots represents cell nucleus.

**Figure 5:**
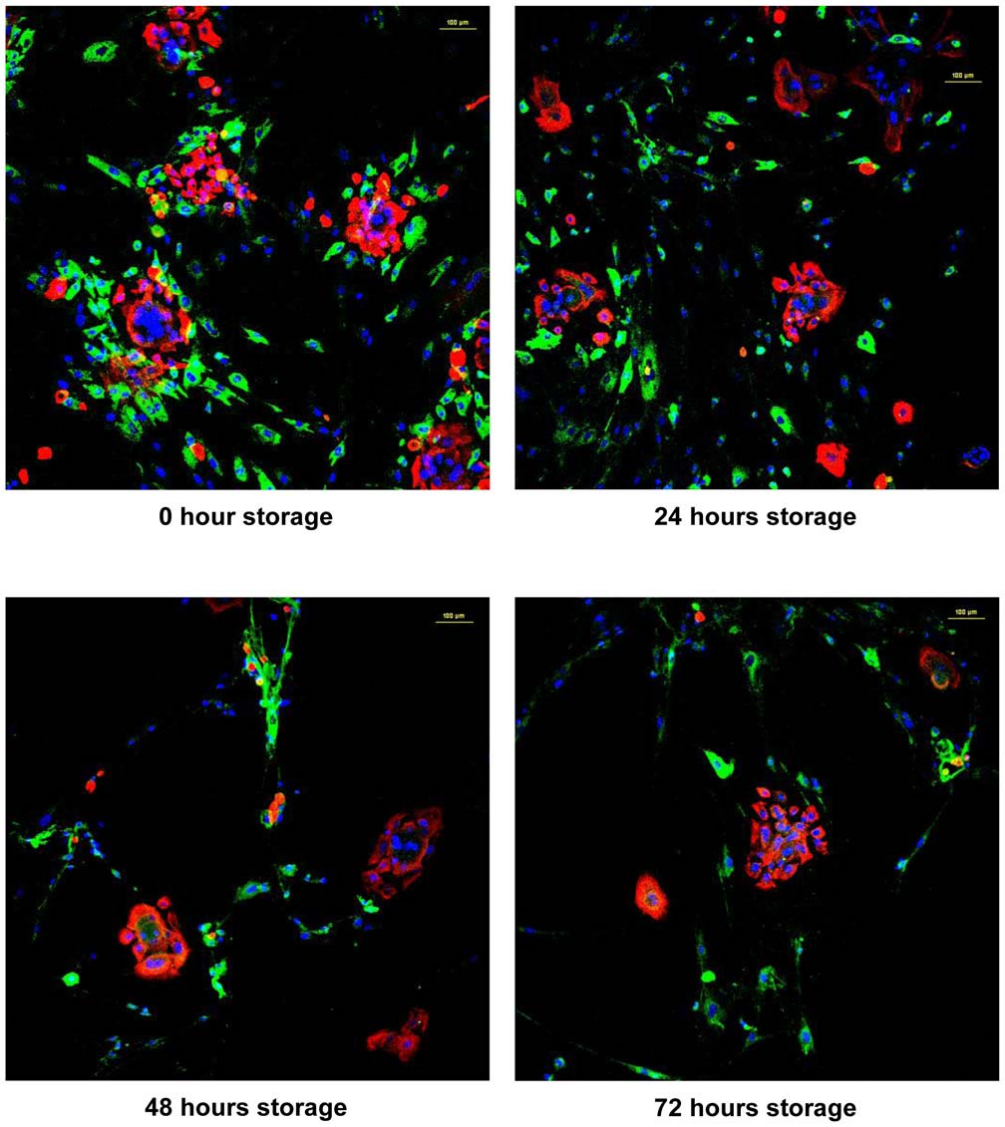
Immunocytochemistry staining of cells in co-culture after liberation from MyDerm™ and cultivation for another 144 h in monolayer culture with Rabbit Anti-Human Polyclonal Collagen III Primary Antibody (green) and Mouse Anti-Human Cytokeratin 14 Monoclonal Antibody (red).

Immunocytochemical staining (Figure 5) using the same set of primary antibodies as above also revealed the presence of both keratinocytes and fibroblasts in the co-culture with keratinocytes being stained fluorescent red and fibroblasts being stained fluorescent green.

### Growth Kinetics of Fibroblasts and Keratinocytes

**Figure 6:**
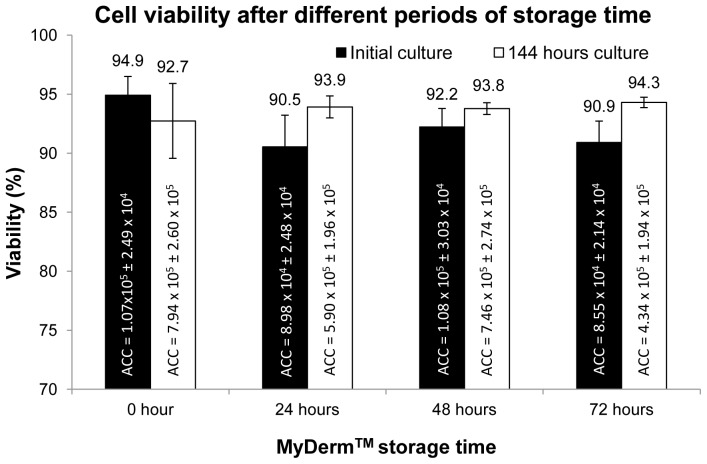
Cell viability at different periods of MyDerm™ storage time after 144 hours culture. ACC = Average Cell Count.

**Figure 7:**
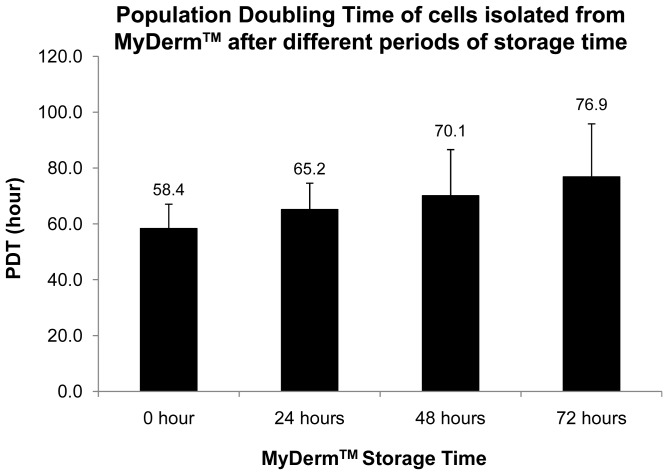
Population doubling time (PDT) of cells at different periods of MyDerm™ storage time after 144 hours culture.

Figure 6 showed the viability of the cells liberated from MyDerm™ (n = 6) after digestion. The viability was 94.9%±1.6% for 0 hour storage, 90.5%±2.7% for 24 hour storage, 92.2%±1.6% for 48 hour storage and 90.9%±1.8% for 72 hour storage groups. There were no significant differences observed between these 4 groups. After 144 hours of monolayer culture, cells were trypsinized and the viability was found to have increased from their respective initial culture except for the 0 hour storage group, although no significant difference was observed (p = 0.06). There was a slight increase in cell viability with increased storage time but was not statistically significant. In addition, population doubling time (PDT) of the cells liberated from MyDerm™ was lowest for 0 hour storage group (58.4±8.7 hours) and gradually increased to 76.9±19 hours for 72 hours storage group as shown in Figure 7. However, no statistically significant differences were found between groups.

### Quantitative Gene Expression Analysis

**Figure 8:**
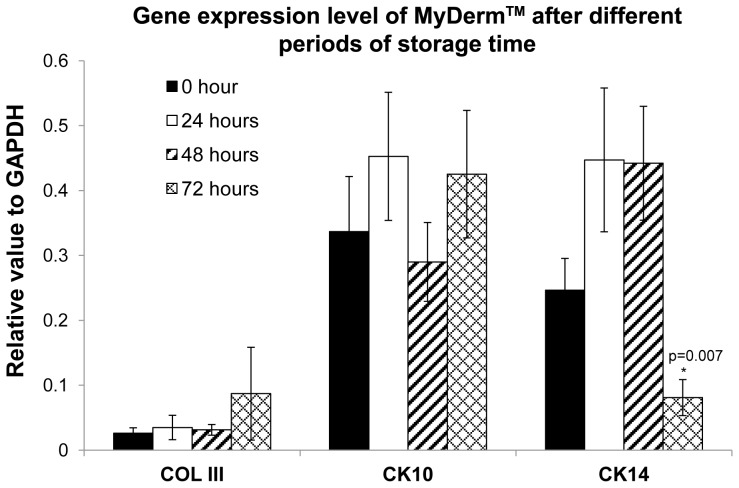
The gene expression levels of cells relative to GAPDH at different periods of MyDerm™ storage time.

Results (Figure 8) showed that MyDerm™ at different storage time had similar levels of expression of COL III gene (0.026±0.008 for 0 hour; 0.035±0.019 for 24 hours; 0.031±0.008 for 48 hours and 0.087±0.071 for 72 hours storage groups) (p = 0.643). The CK10 gene was minimally expressed for 0 hour (0.337±0.085), increased for 24 hours (0.453±0.098), and later dropped for 48 (0.290±0.061) and 72 hours storage groups (0.425±0.098) but no statistically significant differences were observed. The CK14 gene was highly expressed for 0 (0.246±0.049), 24 (0.447±0.111) and 48 hours storage groups (0.042±0.087) and significantly decreased for 72 hours storage group (0.081±0.027) with p = 0.007. However, no significant differences was found between the 0 hour storage group compared to the 72 hours storage group (p = 0.436).

## Discussion

Patients who require skin grafting usually need fast wound closure and healing to minimize the risk of bacterial infection which could be fatal if not treated promptly. Many studies reported that patients treated with skin substitutes, which acted as a temporary biological dressing, had a significantly faster wound closure time compared to their respective control groups. [13], [14], [15], [19], [20]. MyDerm™ is a fully autologous living bilayered skin substitutes that can be integrated completely with the patient's skin after grafting, thus is expected to have comparatively faster wound closer.

The effectiveness of the construct on wound closure depends on the quality of cells in the skin substitutes. For a successful graft uptake like any other organ transplantations, the cells in the construct are to be maintained in a viable state presenting normal phenotype and growth properties prior to grafting. In clinical practice, for some instances, constructs are required to be stored for a certain period time before grafting in order to make allowance for time delays such as transportation, stabilizing patients' conditions and availability of surgeons and facility; thus the demand on evaluation of the quality of the construct after storage for a period of time is important. Considering this fact, the current study was aimed to determine the shelf life of MyDerm™ when stored at 4°C for a maximum of 72 hours by evaluating the quality via analyzing the phenotype (histology of the construct and morphology of cells), growth properties (viability and population doubling time) of cells and the gene expression levels in the construct.

In the *in vivo* system, the survival of the tissue depends on the availability of oxygen and nutrient. Thus, organs harvested for transplantations typically have a short shelf life although maintained in controlled storage conditions, and become unusable once they have exceeded certain storage time. Hearts and lungs need to be transplanted within 5 hours after harvest, livers within 18 hours, pancreas within 20 hours, kidney within 72 hours, corneas within 240 hours, heart valves, skin and bones within 5 years [21]. Besides oxygen and nutrients, tissue survival also depends on cell-cell and cell-matrix interactions and this is especially true for 3D constructs with different cell types and extracellular matrix as support [22], [23].

Since tissue engineered constructs are made of viable cells, they have a limited shelf life. Thus, maintenance of tissue engineered construct in a stable and functional form requires strict conditions such as temperature and nutrient supply. According to Lewis and Sexauer (2006), some tissue engineered products may have a shelf life of 72 hours post-cellular harvest. Beyond this period, the changes in histological feature of the tissue engineered construct as well as the decrease in viability of the cells in the construct can be observed [24]. In the current study, it was found that MyDerm™ stored at 4°C for maximum of 72 hours did not show any significant changes in the histological features of the construct and the morphological property of the cells liberated from the construct. Moreover, cells liberated from the skin construct which was stored at different time periods (maximum of 72 hours) were able to maintain viability of above 90% (on average) and retain their proliferative potential. These results indicated that the construct could be stored for at least 72 hours prior to grafting without significantly compromising the quality of the construct.

Maintenances of functional activity of the cells in the construct demand the continuous supply of oxygen and nutrient. When MyDerm™ was stored in 4°C in pure F12∶DMEM medium, the low temperature would have kept the cells in minimal activity and hence lesser nutrient were required for maintenance, allowing the cells to stay alive for a longer period of time [25]. The skin construct was in the form of a sheet which was submerged in the nutrient medium, thus maximizing the tissue's access to the surrounding nutrient. Furthermore, the tissue engineered skin was constructed with human plasma that contained a mixture of hormones and growth factors which could provide nutrient to maintain the cells in viable state in the construct [26].

When the histology of the bilayered skin construct in our study was examined, it was found that the engineered skin did not show the different layers of a normal epidermis. This was because the keratinocytes were embedded into the fibrin gel where they were scattered by chance without close contact to each other. Under these circumstances, differentiation of epidermis cannot be expected as cell-cell and cell-matrix interactions are essential for this process [22], [23], [27]. Furthermore, the construct was stored immersed in F12∶DMEM medium at 4°C and this condition may not allow the construct to stratify as keratinocytes will only stratify and subsequently cornify if culture under air-liquid interface condition [28], [29].

H&E staining revealed that the morphology of the skin construct remained the same at different storage time points and that MyDerm™ were made up of two layers (Figure 3). Cell nucleus could be clearly seen although it was not possible to differentiate the fibroblasts from the keratinocytes at this stage. The cells in the construct were distributed homogeneously. It also allowed ample space for cell proliferation when grafted onto the patient.

Immunohistochemistry and immunocytochemistry staining with Alexa Fluor® 594 goat anti-mouse IgG (red-fluorescent dye staining on the keratinocytes) and Alexa Fluor® 488 goat anti-rabbit (green-fluorescent dye staining on the fibroblasts) confirmed that MyDerm™ was indeed bilayered (Figure 4) and the cells liberated from MyDerm™ consisted of both cell types and were proliferating after 144 hours of culturing (Figure 5).

Figures 2 and 5 showed that skin cells reduced adherence ability with longer storage duration as the cell density decreased with increasing storage time. The longer the cells were maintained in the 3D skin construct, the lower the efficiency of cell adherence to the culture vessels. This could be due to the lower cell viability (not significant) and 3D living environment adaptation as in the skin construct. This observation was similar to the slow cell adherence when primary skin cells were isolated from native skin, which was also in a 3D living environment. Upon adaptation to monolayer culture, these cells will attach rapidly in subsequent passages.

When the growth kinetics was examined, it was found that the PDTs of our cells were relatively higher compared to primary skin cell culture. This result could be due to the fact that the skin cells have adapted to the 3D living environment in the construct and have reduced in proliferation. In addition, the skin cells in the construct were exposed to human plasma and calcium that might have caused the cells to start differentiating and therefore reduced their proliferative capacity.

Cytokeratins 10 and 14 (CK10 and CK14), a multigene family of polypeptides are believed to be expressed in all keratinocytes and the epidermis of most body locations [30], [31]. Collagen is the most abundant protein and is essential in the contiguous formation of the interstitum through the epidermis. Type I and III collagen (COL III) are formed in human skin primarily in the papillary dermis just beneath the epidermis [32]. In this study, CK10 and CK14 gene were used as a marker for suprabasal and basal keratinocytes respectively whereas the COL III gene was used as a marker for the dermal layer [3], [33]. When gene expression was analyzed, it was found that all three genes expressed by native skin cells were also expressed by the keratinocytes and fibroblasts liberated from MyDerm™ at different storage times. Although there was a significant drop in the CK14 gene level at 72 hours storage when compared to the 48 hours, no significant difference was found when compared to the 0 hour storage. CK14 is a proliferative marker for keratinocytes. The CK14 expression level will drop when keratinocytes are preparing for differentiation. During construct formation, keratinocytes may be stimulated to differentiate when they were exposed to human plasma and CaCl_2_. Hence, the drop of CK14 gene expression also reflects a decrease in the keratinocytes proliferative activities. In addition, CK14 gene expression may have decreased accordingly with increased differentiation at longer storage duration due to the physiological changes of the cells in a 3D environment (construct) compared to monolayer culture.

### Conclusion

This study demonstrated that MyDerm™ can be stored in pure F12∶DMEM medium at 4°C for up to 72 hours and yet showed good viability and population doubling time. The modest drop in cell viability and increased in population doubling time was not a significant concern. The morphology of cells remained unchanged with time and cells could be seen to have a homogenous distribution in histological analysis of the constructs. Gene expression changes for CK10, CK14 and COL III revealed no significant alteration in the patterns of gene expression and were comparable between different storage times. The long shelf life of MyDerm™ enables this skin substitute to be transported to other regions of the country and even to other parts of the world when maintained under good storage conditions.
