# Correction: Shelf-Life Evaluation of Bilayered Human Skin Equivalent, MyDerm™

**DOI:** 10.1371/annotation/44cd1027-1f9e-4843-b013-84ca45ae942f

**Published:** 2012-09-12

**Authors:** Wan Tai Seet, Maarof Manira, Khairoji Khairul Anuar, Kien-Hui Chua, Abdul Wahab Ahmad Irfan, Min Hwei Ng, Bin Saim Aminuddin, Bt Hj Idrus Ruszymah

The second author's name appears incorrectly. The correct name is: Maarof Manira. The correct citation is: Seet WT, Manira M, Khairul Anuar K, Chua K-H, Ahmad Irfan AW, et al. (2012) Shelf-Life Evaluation of Bilayered Human Skin Equivalent, MyDerm™. PLoS ONE 7(8): e40978. doi:10.1371/journal.pone.0040978 

